# Pacing During and Physiological Response After a 12-Hour Ultra-Marathon in a 95-Year-Old Male Runner

**DOI:** 10.3389/fphys.2018.01875

**Published:** 2019-01-04

**Authors:** Beat Knechtle, Zbigniew Jastrzebski, Thomas Rosemann, Pantelis T. Nikolaidis

**Affiliations:** ^1^Medbase St. Gallen Am Vadianplatz, St. Gallen, Switzerland; ^2^Institute of Primary Care, University of Zurich, Zurich, Switzerland; ^3^Department of Tourism and Recreation, Gdańsk University of Physical Education and Sport, Gdańsk, Poland; ^4^Exercise Physiology Laboratory, Nikaia, Greece

**Keywords:** master athlete, elderly, endurance, performance, running

## Abstract

In recent years, outstanding performances of elderly people up to 100 years have been reported. In this case study, pacing during and recovery after a 12-h ultra-marathon were described for a 95-year old runner. The athlete achieved a total distance of 52.987 km. Pacing followed a parabolic pattern (U-shaped), where the speed decreased till the middle of the race and then increased. However, no end spurt was observed. A large main effect of lap quartile on speed was observed, where the second quartile was slower than the first quartile and forth. The smallest variability was shown in the first quartile and the largest in the second quartile. During recovery, erythrocytes, hemoglobin and hematocrit increased whereas thrombocytes and leucocytes decreased. CRP, GOT, GPT, y-GT, CK, and LDH were increased post-race and decreased to reference range during recovery. Also, creatinine and urea decreased during recovery. Creatinine clearance increased during recovery. Sodium increased during recovery and remained constantly within the reference range. During recovery body fat and visceral fat mass decreased, whereas body water and lean body mass increased. In summary, a 95-year-old man was able to run during 12 h using a U-shaped pacing and achieving a total distance of nearly 53 km. Increased selected hematological and biochemical parameters returned to pre-race values within a recovery phase of 5 days.

## Introduction

Ultra-marathon running is of high popularity where recent findings showed an increase in both the number of races (Cejka et al., 2014) and successful finishers (Knechtle et al., 2018b). It is well documented that the increase in the number of successful ultra-marathoners in recent years is due to the increase in both women (Hoffman, 2010) and age group athletes (Knechtle et al., 2014).

While it is well-known that age group marathoners can compete late in their life until the age of 70 years (Jokl et al., 2004; Lepers and Cattagni, 2012), 80 years (Brendle et al., 2003) or even to 90 years (Addison et al., 2015; Ahmadyar et al., 2016), very little is known regarding elderly ultra-marathoners of very high ages (Knechtle and Nikolaidis, 2018a). We know from large data set analyses that elderly runners of 70 years (Rüst et al., 2015) up to 85 years (Rust et al., 2014) are able to finish an ultra-marathon.

Pacing is an important aspect for a successful race finish in marathon (Nikolaidis and Knechtle, 2017a,b, 2018b,c; Diaz et al., 2018) and ultra-marathon (Lambert et al., 2004) running. We recently got new insights in the pacing of master marathoners (Nikolaidis and Knechtle, 2017a,b, 2018b), but very little is known for the pacing of master ultra-marathoners (Knechtle et al., 2015; Rüst et al., 2015; Knechtle and Nikolaidis, 2018a; Knechtle et al., 2018a). In a recent case report pacing and recovery phase of a 94-year-old runner in a 6-h ultra-marathon has been reported (Knechtle and Nikolaidis, 2018a), but no longer ultra-marathon duration has been investigated for an ultra-marathoner of this age. In the present case report we investigate the same runner one year later at the age of 95 years in a 12-hrun.

It is well-known that ultra-marathon running leads to considerable acute changes in biomarkers deviating from reference values in specific organs or organ systems such as skeletal muscles, heart, liver, kidney, immune, and endocrine system (Knechtle and Nikolaidis, 2018b). Usually, these changes are temporary and depend on intensity and duration of the performance, and generally, they normalize within a few days after the race (Knechtle and Nikolaidis, 2018b); thus, they should be considered as acute physiological responses to ultra-endurance exercise rather than pathological.

In this case study we investigated, first, the pacing during a 12-h ultra-marathon in a 95-year-old runner and, second, the recovery phase after the race. Regarding the age group world records in ultra-marathon running^1^ and existing scientific reports, no person at this age ever competed in such a race. Moreover, although the age-related differences in ultra-marathon running performance have been well-documented (Knechtle et al., 2014; Nikolaidis and Knechtle, 2018d), no study has been ever conducted in a 95-year-old runner with regards to the variation of blood physiology during an ultra-marathon race and recovery.

## Materials and Methods

### Ethics Statement

This case report was approved by the ethical review board of Kanton St. Gallen, Switzerland. The runner provided his written informed consent to the analysis and publication of his data.

### The Runner and the Race

Our runner is born in 1923 and started running after his retirement at the age of 65 years. He mainly competed in short distance running races like 5 km, 10 km, and switched later to half-marathon (Knechtle et al., 2010). At the age of 90 years, he successfully completed his first marathon (Mueller et al., 2014). He is the current record holder of the European record in half-marathon and marathon running in age group in M90^2^. In the preparation of this race, he trained 5 days per week on weekdays with a break during the weekend. The daily training varied from 5 to 10 km. One of the training units was together with a group of recreational runners.

Regarding his old age (95 years old), a mild to moderate arterial hypertension is treated with an ACE-inhibitor (angiotensin converting enzyme inhibitor) (5 mg Lisitril^®^) and osteopenia is treated with daily intake of 1000 mg calcium (CALCIUM Sandoz^®^ Brausetabl 1000 mg).

On May 12, 2018, the athlete started in the 12-h run at midnight within the ‘Sri Chinmoy 12 + 24 Stunden-Lauf’ held annually in Basel, Switzerland (Stunden-Lauf, 1988). The course is a flat circuit (1101.4 m) on asphalt, lit at night and officially measured by an IAAF-measurer Grade B. The race has the IAU bronze label since 2007. Lap control consists of an electronic timekeeping (two chips at the back of the race number) plus a personal lap counter at the counting stating and a video camera as backup. After passing each lap the runners have a well visible digital clock. With an electronic chip attached to the race number, the time each lap is measured by an official timekeeping company. When the athlete enters the last lap, he takes a little flag with the starting number on it and leaves it at the final whistle on the edge of the road. The organizer then measures the distance so that the full distance can be measured exactly.

The organizer offers a buffet at the runners each round pastas drinks (e.g., water, tea warm, caffeinated drinks, isotonic sports drink, broth, malt beer, red bull, and coffee) as well as solid foods (e.g., pasta, potatoes, bread with various pads such as cheese or jam, salt brezels, chips, peanuts, bars, cakes, chocolate, biscuits, fruits such as bananas, oranges, watermelons, and grapes). The runners can also arrange a food-stuffing along the route themselves and feed themselves or by accompanying persons. The runner was supported by a personal female support providing him in the beginning of the race in the morning hot coffee and bread with jam. Later during the race he drank Coca Cola^®^ and ate some fruits. The runners can also take individual breaks. The runner made one short break in about the middle of the race.

### Measurements

Before the competition, we measured body mass, percentage of body fat, fat-free mass and percentage of body water using a bioelectrical impedance scale Tanita BC-545 (Tanita, Arlington Heights, IL, United States) to repeat the measurements after the run and for the 5 days after the race. The reliability and validity of this device has recently been shown (Wang and Hui, 2015).

At the same time points, capillary blood samples at a fingertip were drawn. We measured hemoglobin, hematocrit, leukocytes, platelets, C-reactive protein, creatin-kinase, LDH (Lactate dehydrogenase), GPT (glutamate pyruvate transaminase), GOT (aspartate aminotransferase), γ-GT (gamma-glutamyltransferase), creatinine, potassium and sodium. Hematological analysis [erythrocytes, hemoglobin, hematocrit, thrombocytes, mean cell volume (MCV), mean cell hemoglobin (MCH), mean cell hemoglobin concentration (MCHC) and leucocytes] was performed using ABX Micros CRP 200 medical lab (HORIBA Medical, Montpellier, France) and the analysis of the serum parameters using Fuji Dri-Chem 4000i analysis system (FUJIFILM Corporation, Tokyo, Japan). Both laboratory machines undergo regular internal^3^ and external^4^ quality controls. Creatinine clearance was estimated using the Cockcroft and Gault formula (Cockcroft and Gault, 1976).

### Data Analysis

Both graphical and numerical approaches were used to examine the normality of the data, i.e., visual inspection of normal Q-Q plots and the Shapiro-Wilk test, respectively. Accordingly, parametric statistics were applied. The variation of speed by lap was examined using a fourth degree polynomial regression analysis and the relationship between these variables was estimated by coefficient of determination (*R^2^*). Laps were grouped into quartiles, i.e., 1–12, 13–24, 25–36, and 37–48 laps. A repeated measures analysis of variance examined differences in speed among quartiles. 95% confidence intervals (CI) were calculated for mean differences among quartiles. The magnitude of differences was estimated using eta square classified as small (0.010 < η^2^ ≤ 0.059), medium (0.059 < η^2^ ≤ 0.138), and large (η^2^ > 0.138) was used. The relationship among variables was examined using Pearson correlation coefficient r, whose magnitude was evaluated as trivial (*r* < 0.10), small (0.10 ≤*r* < 0.30), moderate (0.30 ≤*r* < 0.50), large (0.50 ≤*r* < 0.70), very large (0.70 ≤*r* < 0.90) and perfect (*r* ≥ 0.90). The acceptable type I error was set at *p* < 0.05.

## Results

### Performance

**FIGURE 1:**
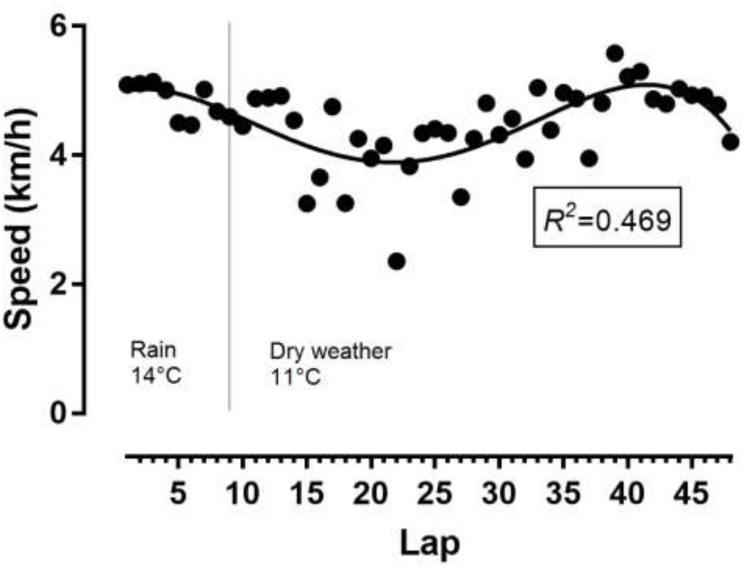
Variation of speed by lap. Temperatures referred to weather conditions during the race. *R^2^*, coefficient of determination.

**FIGURE 2:**
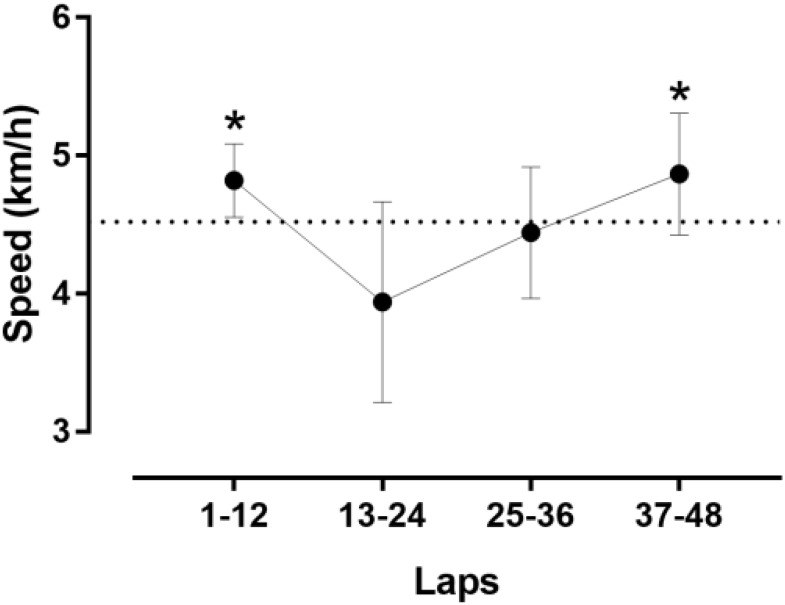
Speed by quartiles of laps.^∗^different from the second quartile at *p* < 0.05. Error bars represent standard deviations.

The athlete achieved a total distance of 52.987 km. As shown in Figure 1, the pacing followed a parabolic pattern (U-shaped), where the speed decreased till the middle of the race and then increased. However, no end spurt was observed. A large main effect of lap quartile on speed was observed (*F*_2,077_ = 8.193, *p* = 0.002, η^2^= 0.427), where the second quartile was slower than the first quartile (-0.88km/h; 95% CI -1.51, -0.25) and forth quartile (-0.93km/h; 95% CI -1.85, 0) (Figure 2). The smallest variability was shown in the first quartile and the largest in the second quartile.

### Laboratory Values Before and After the Race

**FIGURE 3:**
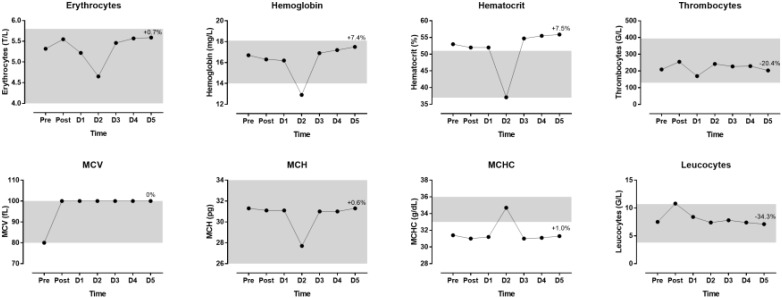
Blood physiology indices during the race and recovery. The shadowed area represents normal reference values. The percentage value over D5 point denotes change from post-race value. D, day post-race.

**FIGURE 4:**
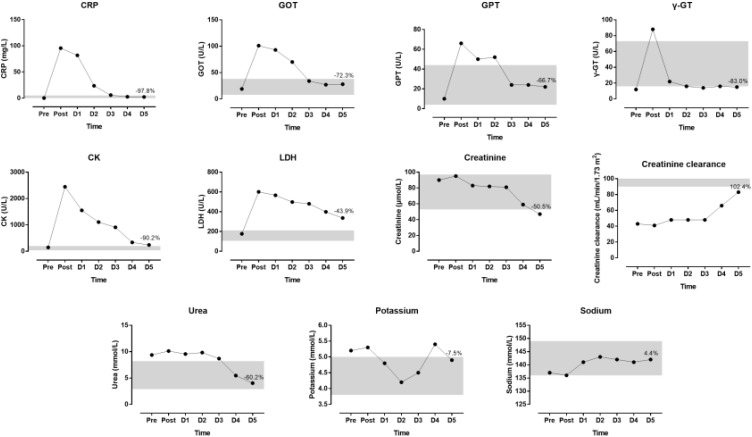
Biochemical indices during the race and recovery. The shadowed area represents normal reference values. The percentage value over D5 point denotes change from post-race value. D, day post-race.

**FIGURE 5:**
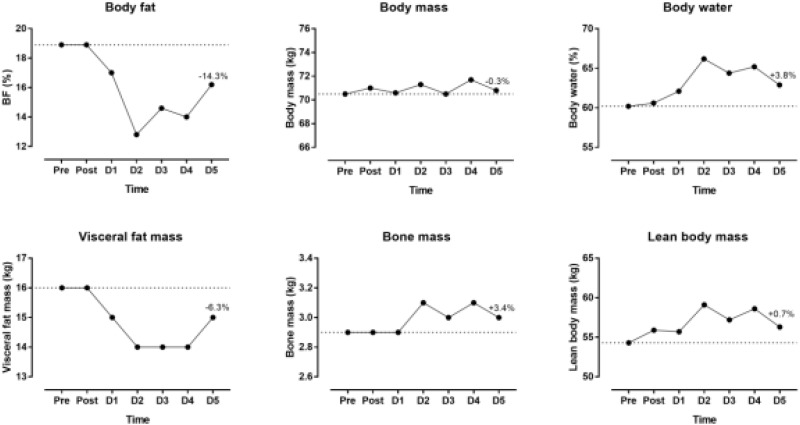
Body composition during the race and recovery. The dashed line represents the pre-race values. The percentage value over D5 point denotes change from post-race value. D, day post-race.

The indices of blood physiology during the race and recovery were presented in Figure 3. Hematocrit and MCHC were out of the normal range. During recovery, erythrocytes, hemoglobin and hematocrit increased whereas thrombocytes and leucocytes decreased. The indices of biochemistry were presented in Figure 4. CRP, GOT, GPT, y-GT, CK, and LDH were increased post-race. During recovery, these variables decreased to normal range where also creatinine and urea decreased. Creatinine clearance increased during recovery. Sodium increased during recovery and remained always within the reference range. Changes in body composition during race and recovery were presented in Figure 5. Post-race, body fat, and visceral fat mass decreased, whereas body water and lean body mass increased.

### Correlations

The change in CRP correlated almost perfectly with the change in CK (*r* = 0.930, *p* = 0.002) and very largely with the change in LDH (*r* = 0.770, *p* = 0.043), whereas the change in Lc correlated almost perfectly with the change in CK (*r* = 0.901, *p* = 0.006), but not with the change in LDH (*r* = 0.632, *p* = 0.128).

The change in body water was not related to the change in hemoglobin (*r* = -0.433, *p* = 0.332) or the change in hematocrit (*r* = -0.436, *p* = 0.328). The change in body water correlated very largely with the change in sodium (*r* = 0.848, *p* = 0.016), but not with the change in creatinine (*r* = -0.421, *p* = 0.347), creatinine clearance (*r* = 0.304, *p* = 0.507), urea (*r* = -0.267, *p* = 0.563), potassium (*r* = -0.565, *p* = 0.186).

The changes in CK and LDH as variables of skeletal muscle damage were not related to the change in creatinine (*r* = 0.615, *p* = 0.142 and *r* = 0.297, *p* = 0.517, respectively) or the change in creatinine clearance (*r* = -0.565, *p* = 0.186 and *r* = -0.302, *p* = 0.510).

## Discussion

In this case report, it was found that (i) a 95-year-old man was able to run during 12 h using a U-shaped pacing and achieving a total distance of nearly 53 km and (ii) increased selected hematological and biochemical parameters returned to pre-race values within a post-race recovery phase of 5 days.

The present ultra-marathoner showed a U-shaped pacing where running speed decreased in the first 6 h to increase in the second 6 h. Furthermore, the second quartile of the run was slower than the first and fourth quartile. Generally, ultra-marathoners show a positive pacing (i.e., slowing down) during the race (Lambert et al., 2004; Hoffman, 2014; Bossi et al., 2017). We might assume that the present ultra-marathoner was aware of his performance in 6-h ultra-marathon running (Knechtle and Nikolaidis, 2018a) and preserved energy for the second half of the race.

The observed physiological responses to the ultra-endurance race were in agreement with the existing literature indicating that regardless of the age of ultra-endurance runners, negative responses of the function of internal organs and skeletal muscles to exercise occurred during the race similarly for younger and older runners (Jastrzebski et al., 2015c). In addition, these responses depended on the length of the distance covered and were greatest at the end of the ultra-endurance exercise.

We found that increased hematological and biochemical parameters returned nearly all to baseline values within the recovery phase of 5 days. It is well-known that biomarkers of skeletal muscle, liver, and kidney damage increase partially dramatically during an ultra-marathon (Jastrzebski et al., 2015a; Shin et al., 2016) where higher age and faster running speeds lead to more pronounced damage in both liver and kidney. However, like in the present case report, all increased values of biomarkers return within a few days to base line values (Kłapcińska et al., 2013; Knechtle and Nikolaidis, 2018b).

An interesting finding was that erythrocytes, hemoglobin, hematocrit and MCH were very low on day 2 where MCHC was increased that day. These changes might be typical for an ultra-marathon and revealed increased rates of destruction of erythrocytes, which was in agreement with the concept of sports anemia. It has been suggested that sports anemia in endurance athletes might be due to hemolysis owing to mechanical trauma and oxidative injuries of erythrocytes (Wu et al., 2004). For instance, this hemolysis might be caused by mechanical trauma when erythrocytes passed through capillaries in contracting muscles and by compression of erythrocytes (e.g., in foot soles during running) (Mairbaurl, 2013). In a 24-h ultra-marathon, significant declines in erythrocytes, hemoglobin and hematocrit were detected 2 days and 9 days after the race (Wu et al., 2004). Leucocytes were increased post-race and returned then to pre-race values. Although the changes in leucocytes during ultra-marathon running might be due to an immune response (Zakovska et al., 2017), the increase in leucocytes after the race is most probably due to an inflammatory process (Jee and Jin, 2012).

Interestingly, total body water was highest that day. Most probably the athlete was much diluted that day. Furthermore, we found an increase in total body water after the race where the increase was not related to changes in biomarkers of renal function but to the increase in sodium. Fluid conservation after an ultra-marathon is well-known (Fellmann et al., 1989) and most likely due to endocrine regulation of plasma sodium concentration (Brge et al., 2011). Indeed, sodium concentration increased during recovery and remained always within the reference range.

During recovery, we found correlations between biomarkers of inflammation (e.g., CRP, Lc) with biomarkers of skeletal muscle damage (e.g., CK, LDH). It should be highlighted that inflammation was indicated by the increase of CRP and leukocytes across race suggesting interplay between hematological (leukocytes) and biochemical (CRP) parameters (Kim et al., 2009). Moreover, it was well-known that biomarkers of both skeletal muscle damage and inflammation increase during an ultra-marathon (Kim et al., 2007). During recovery after an ultra-endurance performance such as an Ironman triathlon, biomarkers of inflammation persisted for 5 days after the race, most likely reflecting incomplete muscle recovery (Neubauer et al., 2008).

The changes in biomarkers of skeletal muscle damage (e.g., CK, LDH) were not related to changes of renal function (e.g., creatinine, creatinine clearance) during recovery. An ultra-marathon often leads to a temporary reduction in renal function (Knechtle and Nikolaidis, 2018b). The prevalence of an acute kidney injury in ultra-marathon running can reach 50% of all runners (Lipman et al., 2017). It is assumed that skeletal muscle damage leads to an increase in muscle proteins (e.g., myoglobin). This increase leads to rhabdomyolysis (Schiff et al., 1978) and, consequently, to renal failure (Uberoi et al., 1991).

One might assume that the increase in selected hematological and biochemical variables might be higher in an older athlete compared to a younger one. The change in laboratory variables seems more dependent upon the length of the performance and the intensity than the age of the runner (Del Coso et al., 2013; Shin et al., 2016). Regarding half-marathon and marathon running, marathon running lead to more pronounced responses in myoglobin, CK-MB-mass, ALT, AST, lactate and phosphate (Niemela et al., 2016). Regarding age, elevations in troponin levels are observed only in young participants (<30 years), most strikingly in those younger than 20 years of age (Niemela et al., 2016). Overall, the responses in the increases in these selected hematological and biochemical variables are in line with other reports (Knechtle and Nikolaidis, 2018b).

The findings of the present case study were of great theoretical and practical value, especially considering the increased participation in ultra-marathon races during the last years (Nikolaidis and Knechtle, 2018a,d). The ability of a 95-year-old man to finish a 12-h-run might be first of all to his adaptive abilities acquired during many years of training. Moreover, the long-term adaptations to ultra-endurance exercise might include the exceptional ability to regulate the intensity of his effort in order not to allow to be interrupted. These relationships were particularly evident from changes in the acid-base balance and lactate concentration in the blood during exercise. Ultra-endurance runners do not allow their body to undergo deep changes, e.g., in saturation or blood acidification by temporarily slowing down the pace of the race. On the other hand, running above their limits would probably interrupt the effort after several minutes as a result of muscle acidification (Jastrzebski et al., 2015b). With regard to the effect of recovery, it is clearly observed that in a healthy person the mechanisms of recovery of organs’ physiological function are efficient regardless of age, which is confirmed by all authors.

Although this was the first study to show that a 95-year-old man was able to run during 12 h, master athletes of very old ages (i.e., 90 years and 100 years) were extending their limits (Lepers and Stapley, 2016) and were able to achieve outstanding performances (Lepers et al., 2016). Future studies would be needed to expand our knowledge about athletic performances of elderly people up to 100 years using larger sample sizes. However, a limitation for full interpretation of the laboratory analyses was the lack of detailed analysis of fluid and food intake during both the race and the recovery period. On the other hand, strength of this study was that it examined the post-race recovery for five consecutive days providing detailed information about the daily variation of blood physiology, whereas most existing research focused on changes during an ultra-marathon race but not on recovery (Zakovska et al., 2017). Furthermore, recovery was previously studied using few post-race assessment days, e.g., the second and ninth post-race day in a 24-h ultra-marathon (Wu et al., 2004) in contrast to the daily assessment of blood physiology for five consecutive post-race days in the present study.

## Conclusion

In conclusion, a 95-year-old man was able to run – despite the fatigue – during 12 h using a U-shaped pacing and achieving a total distance of nearly 53 km. Increased selected hematological and biochemical parameters returned to pre-race values within a post-race recovery phase of 5 days. It seemed that a person at this old age recovered within 5 days from an 12-h ultra-marathon.

## Author Contributions

BK and PN conceived and designed the study. BK collected data. BK, ZJ, TR, and PN analyzed and interpreted the data and drafted the manuscript. BK, ZJ, TR, and PN revised the manuscript and approved the final version.

## Conflict of Interest Statement

The authors declare that the research was conducted in the absence of any commercial or financial relationships that could be construed as a potential conflict of interest.

## Footnotes

1 www.iau-ultramarathon.org/

2 http://european-masters-athletics.org/files/EV_180630.L%C3%85.pdf

3 www.qualab.swiss/Interne-Qualitaetskontrolle.htm

4 www.qualab.swiss/Externe-Qualitaetskontrolle.htm

## References

[B1] Addison O. Steinbrenner G. Goldberg A. P. Katzel L. I. (2015). Aging, fitness, and marathon times in a 91 year-old man who competed in 627 marathons. *Br. J. Med. Med. Res.* 8 1074–1079. 10.9734/bjmmr/2015/17946 26290832 PMC4538980

[B2] Ahmadyar B. Rosemann T. Rüst C. A. Knechtle B. (2016). Improved race times in marathoners older than 75 years in the last 25 years in the world’s largest marathons. *Chin. J. Physiol.* 59 139–147. 10.4077/CJP.2016.BAE382 27188466

[B3] Bossi A. H. Matta G. G. Millet G. Y. Lima P. Pertence L. C. De Lima J. P. (2017). Pacing strategy during 24-hour ultramarathon-distance running. *Int. J. Sports Physiol. Perform.* 12 590–596. 10.1123/ijspp.2016-0237 27618658

[B4] Brendle D. C. Joseph L. J. Sorkin J. D. McNelly D. Katzel L. I. (2003). Aging and marathon times in an 81-year-old man who competed in 591 marathons. *Am. J. Cardiol.* 91 1154–1156. 10.1016/S0002-9149(03)00174-7 12714171

[B5] Brge J. Knechtle B. Knechtle P. Gndinger M. Rst A. C. Rosemann T. (2011). Maintained serum sodium in male ultra-marathoners the role of fluid intake, vasopressin, and aldosterone in fluid and electrolyte regulation. *Horm. Metab. Res.* 43 646–652. 10.1055/s-0031-1284352 21823061

[B6] Cejka N. Rüst C. A. Lepers R. Onywera V. Rosemann T. Knechtle B. (2014). Participation and performance trends in 100-km ultra-marathons worldwide. *J. Sports Sci.* 32 354–366. 10.1080/02640414.2013.825729 24015856

[B7] Cockcroft D. W. Gault M. H. (1976). Prediction of creatinine clearance from serum creatinine. *Nephron* 16 31–41. 10.1159/000180580 1244564

[B8] Del Coso J. Fernandez de Velasco D. Abian-Vicen J. Salinero J. J. Gonzalez-Millan C. Areces F. (2013). Running pace decrease during a marathon is positively related to blood markers of muscle damage. *PLoS One* 8:e57602. 10.1371/journal.pone.0057602 23460881 PMC3583862

[B9] Diaz J. J. Fernandez-Ozcorta E. J. Santos-Concejero J. (2018). The influence of pacing strategy on marathon world records. *Eur. J. Sport Sci.* 18 781–786. 10.1080/17461391.2018.1450899 29557279

[B10] Fellmann N. Bedu M. Giry J. Pharmakis-Amadieu M. Bezou M. J. Barlet J. P. (1989). Hormonal, fluid, and electrolyte changes during a 72-h recovery from a 24-h endurance run. *Int. J. Sports Med.* 10 406–412. 10.1055/s-2007-1024934 2534121

[B11] Hoffman M. D. (2010). Performance trends in 161-km ultramarathons. *Int. J. Sports Med.* 31 31–37. 10.1055/s-0029-1239561 20029736

[B12] Hoffman M. D. (2014). Pacing by winners of a 161-km mountain ultramarathon. *Int. J. Sports Physiol. Perform.* 9 1054–1056. 10.1123/ijspp.2013-0556 24664982

[B13] Jastrzebski Z. Zychowska M. Jastrzêbska M. Prusik K. Prusik K. Kortas J. (2015a). Changes in blood morphology and chosen biochemical parameters in ultra-marathon runners during a 100-km run in relation to the age and speed of runners. *Int. J. Occup. Med. Environ. Health* 29 801–814. 10.13075/ijomeh.1896.00610 27518889

[B14] Jastrzebski Z. Zychowska M. Konieczna A. Ratkowski W. Radziminski L. (2015b). Changes in the acid-base balance and lactate concentration in the blood in amateur ultramarathon runners during a 100-km run. *Biol. Sport* 32 261–265. 10.5604/20831862.1163372 26424931 PMC4577565

[B15] Jastrzebski Z. Zychowska M. Radzimiński Ł Konieczna A. Kortas J. (2015c). Damage to liver and skeletal muscles in marathon runners during a 100 km run with regard to age and running speed. *J. Hum. Kinet.* 45 93–102. 10.1515/hukin-2015-0010 25964813 PMC4415847

[B16] Jee H. Jin Y. (2012). Effects of prolonged endurance exercise on vascular endothelial and inflammation markers. *J. Sports Sci. Med.* 11 719–726. 24150084 PMC3763320

[B17] Jokl P. Sethi P. M. Cooper A. J. (2004). Master’s performance in the New York City Marathon 1983-1999. *Br. J. Sports Med.* 38 408–412. 10.1136/bjsm.2002.003566 15273172 PMC1724857

[B18] Kim H. J. Lee Y. H. Kim C. K. (2007). Biomarkers of muscle and cartilage damage and inflammation during a 200 km run. *Eur. J. Appl. Physiol.* 99 443–447. 10.1007/s00421-006-0362-y 17206443

[B19] Kim H. J. Lee Y. H. Kim C. K. (2009). Changes in serum cartilage oligomeric matrix protein (COMP), plasma CPK and plasma hs-CRP in relation to running distance in a marathon (42.195 km) and an ultra-marathon (200 km) race. *Eur. J. Appl. Physiol.* 105 765–770. 10.1007/s00421-008-0961-x 19125286

[B20] Kłapcińska B. Waåkiewicz Z. Chrapusta S. J. Sadowska-Krêpa E. Czuba M. Langfort J. (2013). Metabolic responses to a 48-h ultra-marathon run in middle-aged male amateur runners. *Eur. J. Appl. Physiol.* 113 2781–2793. 10.1007/s00421-013-2714-8 24002469 PMC3824198

[B21] Knechtle B. Kohler G. Rosemann T. (2010). Study of a European male champion in 10-km road races in the age group > 85 years. *Proc. (Bayl. Univ. Med. Cent.)* 23 259–260. 10.1080/08998280.2010.11928630 20671823 PMC2900979

[B22] Knechtle B. Nikolaidis P. T. (2018a). Pacing in a 94-year-old runner during a 6-hour run. *Open Access J. Sports Med.* 9 19–25. 10.2147/oajsm.s155526 29440939 PMC5804293

[B23] Knechtle B. Nikolaidis P. T. (2018b). Physiology and pathophysiology in ultra-marathon running. *Front. Physiol.* 9:634 10.3389/fphys.2018.00634 PMC5992463 29910741

[B24] Knechtle B. Nikolaidis P. T. Valeri F. (2018a). Russians are the fastest 100-km ultra-marathoners in the world. *PLoS One* 13:e0199701. 10.1371/journal.pone.0199701 29995926 PMC6040753

[B25] Knechtle B. Rosemann T. Nikolaidis P. T. (2018b). Pacing and changes in body composition in 48 h ultra-endurance running-A case study. *Sports* 6:E136. 10.3390/sports6040136 30388759 PMC6315888

[B26] Knechtle B. Rosemann T. Zingg M. A. Stiefel M. Rust C. A. (2015). Pacing strategy in male elite and age group 100 km ultra-marathoners. *Open Access J. Sports Med.* 6 71–80. 10.2147/oajsm.s79568 25848325 PMC4376307

[B27] Knechtle B. Valeri F. Zingg M. A. Rosemann T. Rüst C. A. (2014). What is the age for the fastest ultra-marathon performance in time-limited races from 6 h to 10 days? *Age* 36:9715. 10.1007/s11357-014-9715-3 25280550 PMC4185021

[B28] Lambert M. I. Dugas J. P. Kirkman M. C. Mokone G. G. Waldeck M. R. (2004). Changes in running speeds in a 100 km ultramarathon race. *J. Sports Sci. Med.* 3 167–173. 24482594 PMC3905299

[B29] Lepers R. Cattagni T. (2012). Do older athletes reach limits in their performance during marathon running? *Age* 34 773–781. 10.1007/s11357-011-9271-z 21617894 PMC3337940

[B30] Lepers R. Stapley P. J. (2016). Master athletes are extending the limits of human endurance. *Front. Physiol.* 7:613. 10.3389/fphys.2016.00613 28018241 PMC5149541

[B31] Lepers R. Stapley P. J. Cattagni T. (2016). Centenarian athletes: examples of ultimate human performance? *Age Ageing* 45 729–733. 10.1093/ageing/afw111 27496929

[B32] Lipman G. S. Shea K. Christensen M. Phillips C. Burns P. Higbee R. (2017). Ibuprofen versus placebo effect on acute kidney injury in ultramarathons: a randomised controlled trial. *Emerg. Med. J.* 34 637–642. 10.1136/emermed-2016-206353 28679502

[B33] Mairbaurl H. (2013). Red blood cells in sports: effects of exercise and training on oxygen supply by red blood cells. *Front. Physiol.* 4:332 10.3389/fphys.2013.00332 PMC3824146 24273518

[B34] Mueller S. M. Knechtle B. Knechtle P. Toigo M. (2014). Physiological alterations after a marathon in the first 90-year-old male finisher: case study. *SpringerPlus* 3:608. 10.1186/2193-1801-3-608 25392780 PMC4210455

[B35] Neubauer O. König D. Wagner K. H. (2008). Recovery after an Ironman triathlon: sustained inflammatory responses and muscular stress. *Eur. J. Appl. Physiol.* 104 417–426. 10.1007/s00421-008-0787-6 18548269

[B36] Niemela M. Kangastupa P. Niemela O. Bloigu R. Juvonen T. (2016). Individual responses in biomarkers of health after marathon and half-marathon running: is age a factor in troponin changes? *Scand. J. Clin. Lab. Invest.* 76 575–580. 10.1080/00365513.2016.1225122 27609306

[B37] Nikolaidis P. T. Knechtle B. (2017a). Do fast older runners pace differently from fast younger runners in the ’New York city marathon’? *J. Strength Cond. Res.* 10.1519/jsc.0000000000002159 [Epub ahead of print]. 28746247

[B38] Nikolaidis P. T. Knechtle B. (2017b). Effect of age and performance on pacing of marathon runners. *Open Access J. Sports Med.* 8 171–180. 10.2147/oajsm.s141649 28860876 PMC5571841

[B39] Nikolaidis P. T. Knechtle B. (2018a). Age of peak performance in 50-km ultramarathoners - is it older than in marathoners? *Open Access J. Sports Med.* 9 37–45. 10.2147/oajsm.s154816 29535560 PMC5840300

[B40] Nikolaidis P. T. Knechtle B. (2018b). Pacing in age group marathoners in the “New York City Marathon”. *Res. Sports Med.* 26 86–99. 10.1080/15438627.2017.1393752 29064284

[B41] Nikolaidis P. T. Knechtle B. (2018c). Pacing strategies in the ’athens classic marathon’: physiological and psychological aspects. *Front. Physiol.* 9:1539 10.3389/fphys.2018.01539 PMC6224376 30450055

[B42] Nikolaidis P. T. Knechtle B. (2018d). Performance in 100-km ultra-marathoners - at which age it reaches its peak? *J. Strength Cond. Res.* 10.1519/jsc.0000000000002539 [Epub ahead of print]. 32324710

[B43] Rüst C. A. Rosemann T. Zingg M. A. Knechtle B. (2015). Do non-elite older runners slow down more than younger runners in a 100 km ultra-marathon? *BMC Sports Sci. Med. Rehabil.* 7:1. 10.1186/2052-1847-7-1 25973205 PMC4430021

[B44] Rust C. A. Zingg M. A. Rosemann T. Knechtle B. (2014). Will the age of peak ultra-marathon performance increase with increasing race duration? *BMC Sports Sci. Med. Rehabil.* 6:36. 10.1186/2052-1847-6-36 25337390 PMC4204392

[B45] Schiff H. B. McSearraigh E. T. M. Kallmeyer J. C. Schiff H. B. McSearraigh E. T. M. Kallmeyer J. C. (1978). Myoglobinuria, rhabdomyolysis and marathon running. *QJM* 47 463–472. 751088

[B46] Shin K. A. Park K. D. Ahn J. Park Y. Kim Y. J. (2016). Comparison of changes in biochemical markers for skeletal muscles, hepatic metabolism, and renal function after three types of long-distance running. *Medicine* 95:e3657. 10.1097/MD.0000000000003657 27196469 PMC4902411

[B47] Stunden-Lauf. (1988). *Sri Chinmoy Marathon Teamfrom.* Available at: https://ch.srichinmoyraces.org/self-transcendence-1224-stunden-lauf-basel.

[B48] Uberoi H. S. Dugal J. S. Kasthuri A. S. Kolhe V. S. Kumar A. K. Cruz S. A. (1991). Acute renal failure in severe exertional rhabdomyolysis. *J. Assoc. Phys. India* 39 677–679. 1814900

[B49] Wang L. Hui S. S. (2015). Validity of four commercial bioelectrical impedance scales in measuring body fat among chinese children and adolescents. *Biomed. Res. Int.* 2015:614858. 10.1155/2015/614858 26167491 PMC4475745

[B50] Wu H. J. Chen K. T. Shee B. W. Chang H. C. Huang Y. J. Yang R. S. (2004). Effects of 24 h ultra-marathon on biochemical and hematological parameters. *World J. Gastroenterol.* 10 2711–2714. 10.3748/wjg.v10.i18.2711 15309724 PMC4572198

[B51] Zakovska A. Knechtle B. Chlibkova D. Milickova M. Rosemann T. Nikolaidis P. T. (2017). The effect of a 100-km ultra-marathon under freezing conditions on selected immunological and hematological parameters. *Front. Physiol.* 8:638. 10.3389/fphys.2017.00638 28955243 PMC5600930

